# Effectiveness of an Innovative Mobile-Based Perioperative Care Program for Women Undergoing Breast Cancer Surgery (iCareBreast): Randomized Controlled Trial

**DOI:** 10.2196/71684

**Published:** 2025-04-21

**Authors:** Yan Pang, Honggu He, Ruey-Pyng Ng, Nicole Kim Luan Lee, Me Me Win Htein, Xiao-Xin Zhao, Ying-Hong Li, Elizabeth Jiahui Chan, Lixia Zhu, Guang Yu Liu, Minna Pikkarainen, Swee-Ho Lim

**Affiliations:** 1 Alice Lee Centre for Nursing Studies Yong Loo Lin School of Medicine National University of Singapore Singapore Singapore; 2 National University Health System Singapore Singapore; 3 KK Women’s and Children’s Hospital Singapore Singapore; 4 Digitalization of Healthcare Services Oslomet, Oslo Metropolitan University Oslo Norway; 5 Martti Ahtisaari Institute University of Oulu Oulu Finland

**Keywords:** breast cancer, digital health, mHealth, mobile health, psychosocial, randomized controlled trial, self-efficacy, mobile phone

## Abstract

**Background:**

Breast cancer is one of the most prevalent cancers among women and significantly impacts psychological well-being and health-related quality of life (HR-QoL) during the perioperative period. Mobile health interventions offer a promising approach to providing education and psychosocial support, yet their effectiveness in this context remains underexplored.

**Objective:**

This study aimed to develop and evaluate the effectiveness of an innovative, mobile-based, perioperative care program for women undergoing breast cancer surgery (iCareBreast). The assessment focused on perioperative self-efficacy, anxiety, depression, fatigue, HR-QoL, and perioperative care satisfaction.

**Methods:**

A two-group randomized control trial was conducted at a tertiary hospital in Singapore. The intervention group used the iCareBreast app, offering four main resources: perioperative care guidance, breast cancer and surgery education, psychological support, and social support. The control group received standard hospital care. Participants in the intervention group engaged with the fully automated app daily for 29 days (two weeks before surgery, on the day of surgery, and two weeks after surgery). Data were collected face-to-face or on the web at three time points: baseline, immediately after the intervention (T1; two weeks after surgery), and at a 2.5-month follow-up (T2; three months after surgery). The primary outcome was perioperative care self-efficacy, while secondary outcomes included anxiety, depression, fatigue, HR-QoL, and perioperative care satisfaction.

**Results:**

A total of 123 patients with early-stage breast cancer scheduled for breast surgery were enrolled in the study, with 62 patients assigned to the iCareBreast group and 61 patients to the control group. The results showed no significant differences between the groups in the primary outcome—perioperative self-efficacy—at any time point. Baseline scores were similar (*P*=.80), and while the iCareBreast group showed slightly lower scores at T1 (mean difference [MD] –1.63, 95% CI –3.43 to 0.18; *P*=.08) and T2 (MD –1.90, 95% CI –4.06 to 0.26; *P*=.09), the differences were not statistically significant. Similarly, secondary outcomes, including anxiety, depression, fatigue, HR-QoL, and perioperative care satisfaction, showed no significant changes between groups (all *P*>.05). However, the iCareBreast group reported higher perioperative care satisfaction during the postintervention assessment. Satisfaction scores were comparable at T1 (*P*=.68), while at T2, the iCareBreast group showed a slight increase compared to the control group (MD 0.35, 95% CI 0.04-0.73; *P*=.08), though the difference was not statistically significant.

**Conclusions:**

The mobile-based psychosocial intervention, although satisfied by users, did not demonstrate significant benefits compared to standard care. This highlights the need to refine the iCareBreast app in future iterations to enhance its effectiveness in addressing the targeted health outcomes. Future mobile health research should prioritize optimizing user engagement strategies and incorporating personalized approaches to better address the perioperative care needs of patients with breast cancer.

**Trial Registration:**

ClinicalTrials.gov NCT04172350; https://clinicaltrials.gov/study/NCT04172350

## Introduction

### Background

Breast cancer is the most prevalent cancer among women worldwide, affecting 2.3 million women annually. In 2022, approximately 670,000 women died from the disease, and the incidence of breast cancer continues to rise [1]. In line with global trends, breast cancer is the leading cause of cancer diagnosis and death among women in Singapore [2].

A breast cancer diagnosis and its treatment often bring significant physical challenges, such as fatigue, alongside psychosocial issues like anxiety, depression, and reduced social interactions [3-8]. The perioperative period, which includes the time before and after surgery, is particularly critical, as patients commonly experience heightened fear and anxiety following diagnosis. This anxiety often intensifies in the presurgery phase [9-11], largely due to a lack of knowledge and resulting concerns about treatment outcomes and changes to body image [12,13]. If left unaddressed, these issues can lead to a decline in health-related quality of life (HR-QoL), particularly during key transition and adjustment periods [14,15].

Given the high prevalence of breast cancer, increasing survival rates, and the cancer impact on psychological well-being and HR-QoL, various psychosocial interventions have been developed to support women in coping with the illness. Evidence suggests that effective psychosocial interventions can reduce psychological distress and facilitate adjustment by enhancing cancer-related knowledge, coping skills, and emotional expression [16]. Various psychosocial interventions, such as behavioral cancer stress management, supportive-expressive therapy, meaning-centered psychotherapy, mindfulness-based interventions, acceptance and commitment therapy, behavioral lifestyle programs, yoga, couples-focused therapies, and psychoeducation, have demonstrated significant benefits. These approaches have been shown to alleviate anxiety and depression; improve quality of life; and address mood disturbances, body image concerns, self-esteem, and sexual functioning among patients with breast cancer after surgery [17] and those with early-stage breast cancer undergoing or having completed adjuvant therapy [18].

With advancements in technology and the widespread adoption of smart devices, numerous mobile-based intervention programs have emerged for patients. Studies have reported high satisfaction levels among participants using breast cancer supportive care interventions delivered via eHealth platforms [19]. These eHealth interventions typically offer features such as depression screening [20], medication adherence records [21], symptom reporting [22,23], nutritional advice [24], and self-reporting of sleep patterns [25]. More recently, self-management apps tailored for patients with breast cancer have been developed to enhance quality of life by reducing symptom burdens. These apps provide psychosocial support, coping strategies for side effects, stress management, access to social support networks, breast cancer–related education, and communication channels with health care professionals [26-28]. However, most of these apps are used primarily by patients who have completed active treatment, and the evidence of their effectiveness remains inconclusive.

While existing literature underscores the benefits of psychosocial interventions for patients at specific stages of treatment, most studies focus on patients with breast cancer who have completed active treatment, leaving a gap in addressing the unmet needs of patients in the perioperative phase. Additionally, traditional psychosocial interventions are typically delivered face-to-face in group settings by health care professionals, increasing the workload for hospital staff and limiting the comprehensive delivery of information within a single session. Limited research has explored mobile technology as a platform for delivering psychological interventions, despite its potential to provide timely information and high accessibility. Thus, there is a critical need to develop an innovative, mobile-based, care improvement program tailored to the needs of women undergoing breast cancer surgery, addressing both physical and psychosocial challenges throughout their perioperative journey.

### Aims and Hypothesis

This study aims to evaluate the effectiveness of the newly developed mobile app, iCareBreast, on the health outcomes of women undergoing breast cancer surgery. The hypothesis is that, compared to the control group receiving standard care, participants using the iCareBreast app will report significantly higher levels of perioperative self-efficacy, which serves as the primary outcome. Additionally, the intervention group is expected to report lower levels of anxiety and depression, reduced postoperative fatigue, and an overall improvement in quality of life. Furthermore, we hypothesize that they will express greater satisfaction with their perioperative care compared to those in the control group.

## Methods

### Study Design

This study used a single-center, two-group, pre- and posttest, randomized controlled trial (RCT) design. Participants were randomly assigned to either the intervention group, which received routine hospital care plus the iCareBreast app, or the control group, which received routine hospital care alone, using block randomization. The study aimed to investigate the effectiveness of iCareBreast on the health outcomes of women newly diagnosed with breast cancer and requiring surgical intervention. Outcomes were measured at three time points: baseline (T0), immediately after the intervention or 2 weeks after surgery (T1), and 2.5 months after the intervention or 3 months after surgery (T2). The study was registered with ClinicalTrials.gov (NCT04172350) before commencement, and data were collected from November 2020 to January 2023. The findings are reported in accordance with CONSORT-EHEALTH guidelines (Multimedia Appendix 1).

### Recruitment

Due to limited human resources, the study was conducted at a single site to ensure effective intervention delivery and data collection. Participants were recruited through consecutive sampling at a surgical breast clinic in one of Singapore’s largest public tertiary hospitals, KK Women’s and Children’s Hospital, which provides comprehensive breast cancer care. Participants who were women older than 21 years diagnosed with breast cancer and scheduled for breast cancer surgery, were able to speak either English or Chinese, and had access to a smartphone were enrolled, and those with psychiatric illness, impaired cognitive function, alcohol or substance abuse history, anxiety, or other mood disorder or had been in the bereavement period in the past 6 months were excluded.

### Sample Size Calculation and Determination

A power analysis was conducted to determine the required sample size for this study. Based on an expected difference of 4.5 (SD 7.5) [29] in perioperative self-efficacy scores (primary outcome) between groups, a minimum sample size of 45 per group was necessary to achieve 80% power at a significance level of .05 (2-sided). Accounting for an anticipated 20% dropout rate, a minimum of 112 participants (56 per group) was required. However, early recruitment and data collection experiences indicated a higher-than-expected dropout rate. To accommodate this, an estimated total of 124 participants (62 per group) was deemed sufficient for the study.

### Randomization, Allocation Concealment, and Blinding

To ensure balanced allocation between the intervention and control groups, block randomization with a block size of four was performed using a web-based randomization tool. A blinded study team member (HH) generated the sequences and prepared opaque, sealed envelopes containing the assigned group. A designated research assistant unaware of the block size opened the envelopes in front of participants to reveal their assignment [30,31].

### Intervention

#### Overview

Participants in the control group received routine care, while those in the intervention group received additional support through the iCareBreast mobile app. The app consists of three main components: (1) an individual Zoom (Zoom Video Communications Inc) or in-person session to guide participants in installing the app via the Play Store (Android) or App Store (iOS) and familiarizing them with its functions, accompanied by a brief manual; (2) access to the iCareBreast app from recruitment until two weeks after surgery; and (3) a researcher-accessible dashboard to monitor information access, respond to queries, and send reminders. All data were encrypted and synchronized to Amazon Cloud.

#### iCareBreast App

The iCareBreast app is a digital tool designed to support patients through their perioperative journey, providing care coordination and engagement for surgery preparation and recovery. The app includes four key functions: (1) educational content: information on breast cancer, including presurgery and postsurgery education on topics such as anatomy, treatment options, and anesthesia; (2) perioperative care guidance: step-by-step instructions for the perioperative period, covering preoperative preparation, day-of-surgery guidance, postoperative care (eg, wound and drain management, pain management, and infection monitoring), physiotherapy, and discharge planning; (3) psychological support: daily motivational quotes, mindfulness exercises, and stories from patients with breast cancer; and (4) social support: summarized information on breast cancer support networks available in Singapore.

**Figure 1:**
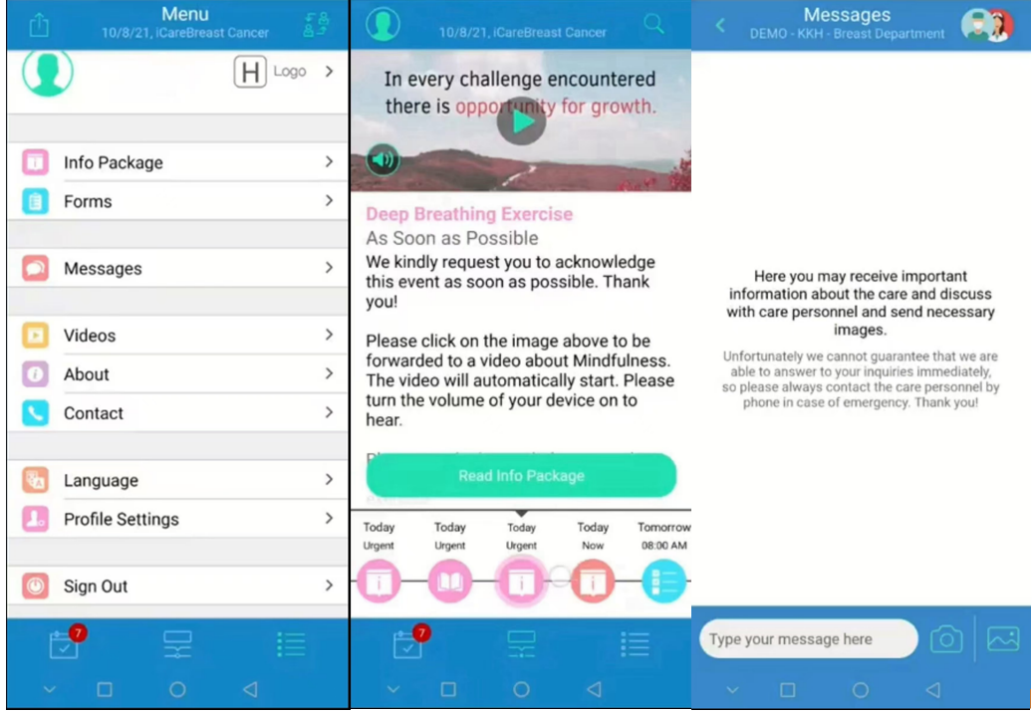
Screenshots of iCareBreast (English version).

The app’s content was developed based on Bandura’s [32] self-efficacy theory, the surgical pathway, hospital physiotherapy protocols, social support components, and positive psychology and mindfulness principles from previous studies conducted by the researchers [33-38]. An expert panel including surgeons, nurses, physiotherapists, and researchers validated the content. The app is designed to be user-friendly, featuring simple, layperson-friendly language, illustrations, animations, videos, and reminders. Screenshots of the app are shown in Figure 1.

Participants in the intervention group were instructed to download the app from the Google Play Store (for Android users) or Apple App Store (for iOS users) and were provided with a unique activation code to log in to allow each participant secure access to the app, ensuring confidentiality. The app was provided free of charge during the study period, and each participant received a user manual. The app enabled a tailored schedule based on each participant’s actual surgery date. After entering the scheduled surgery date, the app delivered daily information throughout the 29-day intervention period (14 days before surgery, on the day of surgery, and 14 days after surgery). The advantage of the tasks is that participants will not get an overflow of instructions at one point but get guidance step by step at the right moment. Participants who joined the study within 1-14 days before surgery were able to review all previous information upon logging in. For instance, if a participant enrolled 5 days before surgery (on day 9 of the preoperative sequence in the app), they received daily content starting from day 9 but were instructed to review content from days 1 to 8 at their convenience. The app sent daily notifications prompting participants to review content and complete specific tasks. Participants acknowledged task completion in the app. The timeline events were color coded as follows: tasks marked in green indicated successfully read and completed daily tasks, while pink indicated tasks that were overdue and awaiting acknowledgment. Events in light blue represented information not yet due for acknowledgment, though they could be read in advance. A summary of the day-to-day content provided to each participant in the intervention group is presented in Multimedia Appendix 2.

Additionally, participants could ask questions through the app’s messaging system, which was managed by health care professionals and researchers.

#### Web-Based Dashboard Management

**Figure 2:**
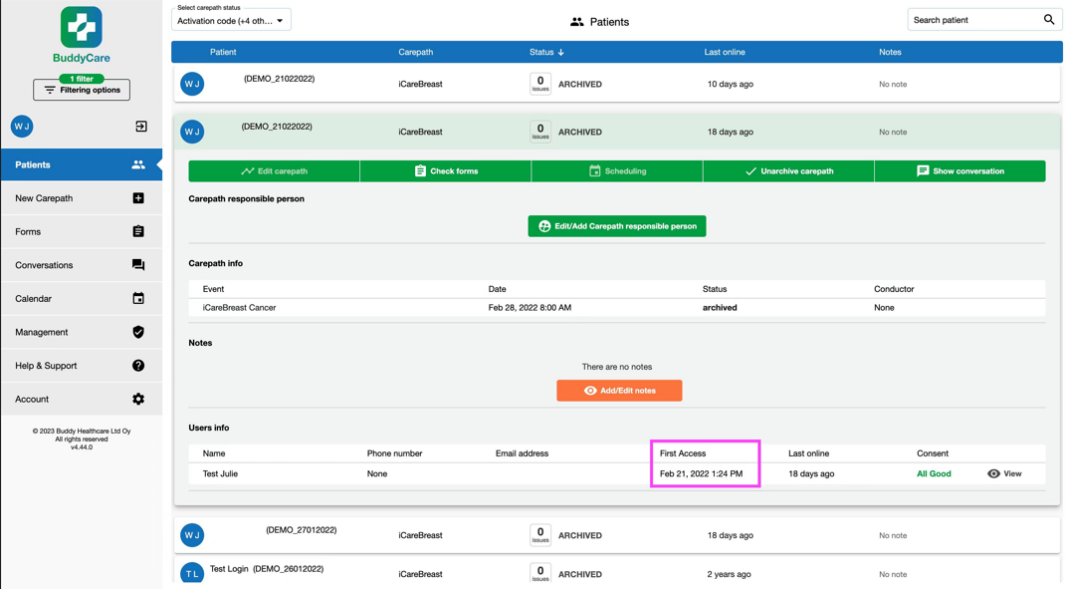
Screenshot of the web-based dashboard of iCareBreast.

Through the web-based dashboard, health care professionals and researchers monitored participants’ app log-in activity and tracked which information had been accessed. Reminders were sent to participants if a daily task was marked as incomplete, with a maximum of two reminders issued through the dashboard. The dashboard also allowed health care professionals and researchers to respond to participants’ queries directly. Figure 2 illustrates the web-based dashboard of the iCareBreast mobile app.

#### Security, Confidentiality, and Technical Support

A notice in the app reminded participants, “Please do not disclose any personal information when using the messaging system,” to prevent the sharing of private identifiable information. All data, including surgery dates and messages entered through the messaging system, were encrypted and synchronized to a secure, web-connected portal on Amazon Cloud. Technical support was provided by the Buddy Healthcare team throughout the study.

#### Control Group

Participants in the control group received the standard of care provided by the treating hospital. This included routine tests; face-to-face education from the surgeon and breast care nurses on treatment information; and postoperative care, such as wound and drain management, supplemented by an educational pamphlet. Physiotherapy exercises were taught during the hospital stay, and patients received a pamphlet with instructions to continue these exercises at home. Psychosocial support was offered through the hospital’s Breast Cancer Support Group, and emotional support was provided by health care professionals.

### Data Collection Procedure

Potential participants were approached during their routine clinic consultations. Those scheduled for breast surgery were initially approached by the attending physician or breast cancer nurses via a study flyer. Participants who expressed interest were then introduced to the research assistant, who provided further details about the study. Consent was obtained either face-to-face or remotely, depending on local hospital policies during the COVID-19 period.

Once informed consent was obtained, participants completed the baseline (T0) assessment, which included a sociodemographic datasheet and questionnaires on health outcomes. After baseline data collection, participants were randomly assigned into either the intervention or control group.

Data collection occurred at two time points during the follow-up period: immediately after the intervention or 2 weeks after surgery (T1), and 2.5 months after the intervention or 3 months after surgery (T2). At T2, a clinical datasheet was completed by the researcher based on each participant’s electronic medical record, documenting the length of hospital stay, type of surgery, and whether chemotherapy or radiotherapy was administered. Data collection at all points of all time was conducted via hard copy or web-based survey, depending on the COVID-19 situation and participant preference.

### Treatment Fidelity

All participants received the same intervention delivered via the mobile app iCareBreast using a standardized instruction manual. A tracker was installed in the iCareBreast app to record the participants’ app content accessing activities. The app also sent automatic daily push notifications to participants encouraging them to use the app and an acknowledgement was required from participants after completing the daily task. The researcher monitored the dashboard and sent reminders to participants that an acknowledgment should not be completed. These measures ensured treatment fidelity [39,40].

### Outcome Measure and Instruments

#### Overview

The primary outcome (perioperative self-efficacy) and secondary outcomes (anxiety, depression, cancer-related fatigue, breast cancer HR-QoL, and perioperative care satisfaction) were assessed using self-reported questionnaires. Both the English and Chinese versions of the questionnaires used in this study are reliable and have been locally validated.

#### Perioperative Self-Efficacy

Perioperative self-efficacy was measured using the 10-item General Self-Efficacy Scale [41]. The total General Self-Efficacy Scale score is calculated by summing the scores of all items, with a range from 10 to 40. A higher score indicates a higher level of self-efficacy. The internal consistency of the English version is between 0.76 and 0.90 [41], while the Chinese version has a high internal consistency of 0.95 [42].

#### Anxiety and Depression

Anxiety and depression levels were measured using the 14-item Hospital Anxiety and Depression Scale [43], which contains 7 items for anxiety and 7 items for depression. Each item is scored on a 4-point scale (0-3), resulting in possible scores ranging from 0 to 21 for both anxiety and depression. Scores of 8-10 on each scale may suggest borderline risk of anxiety or depression, while scores of 11 or higher indicate probable severe anxiety or depression. Cronbach α values for the Hospital Anxiety and Depression Scale range from 0.68 to 0.93 for anxiety and 0.67 to 0.90 for depression [43]. The Chinese version shows Cronbach α values ≥0.84 for both anxiety and depression [44].

#### Fatigue

Fatigue was measured using the 30-item Multidimensional Fatigue Symptom Inventory-Short Form [45]. Each item is rated on a 5-point Likert scale from 0=not at all to 4=extremely. Scores are summed to obtain scores for five subscales: general fatigue, physical fatigue, emotional fatigue, mental fatigue, and vigor. The total fatigue score is calculated by summing the first four subscales (general, physical, emotional, and mental fatigue) and subtracting the vigor score. Total scores range from –24 to 96, with higher scores indicating higher levels of fatigue. Both the English and Chinese versions have been locally validated in patients with breast cancer and lymphoma, demonstrating high internal consistency (α=0.749 to 0.944) [45].

#### Breast Cancer Quality of Life

Quality of life was measured using the 23-item European Organisation for Research and Treatment of Cancer Quality of Life Questionnaire—Breast Cancer Module scale [46], which uses Likert scales ranging from 1=not at all to 4=very much. The items are grouped according to subscales, with a specific recall period for each. The raw scores for each subscale and single item are linearly converted to a 0-100 scale. For functional scales and single items (eg, body image, sexuality, and future perspective), higher scores indicate better function. In contrast, for symptom scales, higher scores reflect worse symptom levels. To prevent respondent fatigue, a shortened version of the scale was used [47]. The European Organisation for Research and Treatment of Cancer Quality of Life Questionnaire—Breast Cancer Module has been locally validated, showing good internal consistency with Cronbach α of 0.873 for both the English and Chinese versions [48].

#### Perioperative Satisfaction

A 6-point Ordinal Descriptive Scale was used to assess participants’ self-reported satisfaction with the perioperative care they received. This scale has been used by the corresponding author in multiple previous studies and was chosen for its simplicity and ease of use.

### Ethical Considerations

Ethical approval was obtained from the local ethics board (SingHealth Centralised Institutional Review Board [CIRB reference: 2019/2632]). All participants provided informed consent in either the English version or a fully translated Simplified Chinese version, in accordance with the principles of the Helsinki Declaration. Participants’ identities were anonymized, and they were randomly assigned to either the intervention or control group. Participation in the study was voluntary, and no compensation was provided.

### Statistical Analysis

Statistical analyses were conducted using SPSS (version 29.0; IBM Corp), with statistical significance set at *P*<.05. An intention-to-treat approach was applied throughout the analysis. Prior to conducting the main analyses, the Little missing-completely-at-random test was used, confirming that missing data were random across all variables in the dataset.

Descriptive statistics were presented as n (%) for categorical data and mean (SD) for continuous data. The normality of continuous variables was assessed using the Shapiro-Wilk test. Three variables that were not normally distributed, the Mann-Whitney *U* test was used instead of the 2-sided *t* test.

Chi-square tests, and where appropriate, independent samples 2-sided *t* tests or 2-sided Mann-Whitney *U* tests, were used to compare sociodemographic and clinical characteristics, as well as baseline outcome differences, between the intervention and control groups.

Independent samples *t* tests or Mann-Whitney *U* tests, as appropriate, were used to assess differences between the intervention and control groups in posttests 1 and 2. A linear mixed model for repeated measurements, with participant ID as a random factor, was used to assess the intervention effect on outcomes over time. The main effects for group, time, and the group×time interaction were examined. A subgroup analysis using a linear mixed model, including a time×engagement level interaction, was conducted to compare changes in the primary outcome over time between high and low-app engagement groups. Additionally, subgroup analyses were performed using independent samples *t* tests or Mann-Whitney *U* tests to compare primary and secondary outcomes between participants with high and low app engagement levels at follow-up time points. To control for type I errors from multiple comparisons, Bonferroni correction was applied to independent *t* tests and Mann-Whitney *U* tests, while SPSS automatically applied it for pairwise comparisons in the linear mixed model where applicable. Statistical significance was set at *P*<.05 (2-sided).

## Results

### Participants

**Figure 3:**
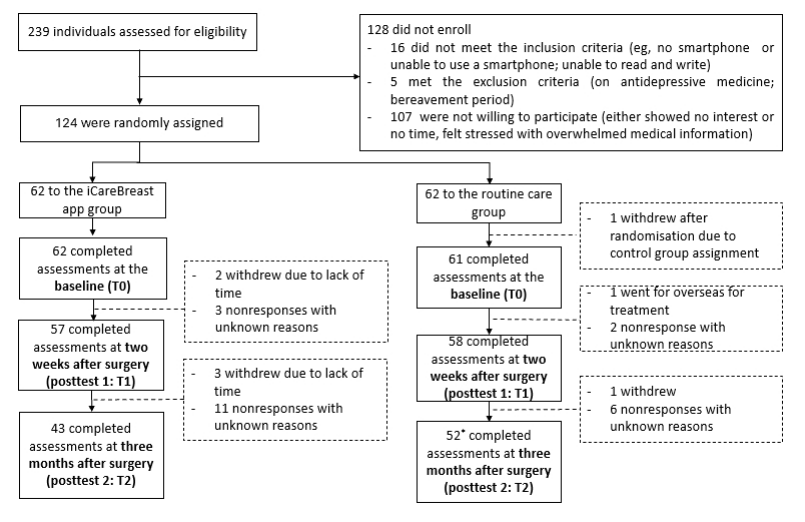
CONSORT (Consolidated Standards of Reporting Trials) diagram. *One participant did not complete T1, but went on to complete T2.

A total of 124 women were enrolled and randomly assigned to two groups, with 62 participants in each group, from November 2020 to January 2023. All follow-up data were collected by January 2023. One participant in the control group withdrew after randomization due to dissatisfaction with the assignment. Her baseline survey was only partially completed, so the research team formally withdrew her from the study. At T1, a total of 8% (5/62) of participants in the intervention group and 5% (3/61) of participants in the control group dropped out. At T2, a total of 25% (14/57) of participants in the intervention group and 12% (7/59) of participants in the control group dropped out; notably, 1 participant in the intervention group who missed T1 completed T2. Consequently, 123 participants were included in the final analysis. There were no significant differences between groups in dropout rates or the timing of the last completed assessment, suggesting that attrition likely did not bias treatment effects. The study’s CONSORT (Consolidated Standards of Reporting Trials) diagram is shown in Figure 3.

### Overview

#### Comparison of the Sociodemographic and Clinical Variables of the Participants, as Well as Baseline Outcomes Between the Two Groups

Table 1 presents the sociodemographic and clinical characteristics of newly diagnosed patients with breast cancer undergoing breast surgery at T0. The participants had a mean age of 55.99 (SD 11.27) years, a mean hospital stay of 2.59 (SD 2.13) days, and a mean time from diagnosis to surgery of 84.06 (SD 84.69) days. The majority of participants were Chinese (93/123, 75.6%) and married (88/123, 71.5%). Slightly more than half of the patients were college or university graduates (62/123, 50.4%). Over half of the patients were employed (75/123, 61%) and had a monthly income exceeding SGD $3000 (approximately US $2276.28; 67/123, 54.5%). Approximately 80.5% (99/123) were diagnosed with invasive breast cancer (stages 1-3), and nearly 93.5% (115/123) had undergone either mastectomy or lumpectomy. Nearly half of the participants, 48% (59/123), received chemotherapy, and one-third (41/123) of participants received radiotherapy. Further details on the sociodemographic and clinical characteristics of both groups are provided in Table 1. Results from chi-square tests, independent sample *t* tests, and Mann-Whitney *U* tests showed no significant differences in sociodemographic or clinical characteristics at baseline between the groups (Table 1), which supported successful randomization. Additionally, Table 2 indicates no statistically significant differences in baseline outcomes for perioperative self-efficacy, depression, anxiety, fatigue, and overall quality of life.

**Table 1:** Comparisons of the participants’ sociodemographic characteristics and clinical data between the two groups (n=123).

Sociodemographic characteristics and clinical data		iCareBreast group (n=62)	Control group (n=61)	Chi-square (*df*), *t* test (*df*), or Fisher exact test		*P* value	
Age (years), mean (SD)		56.6 (11.7)	55.4 (10.9)	0.61 (121)		.54^a^	
Hospital stay length (days), mean (SD)		2.4 (2.0)	2.8 (2.2)	—^b^		.18^c^	
Diagnosis to operation length (days), mean, (SD)		71.8 (69.8)	96.6 (91.1)	—		.31^c^	
**Marital status, n (%)**					0.30 (2)		.86^d^
	Married	43 (69.3)	45 (73.8)				
	Single	13 (21)	11 (18)				
	Divorced or separated	6 (9.7)	5 (8.2)				
**Ethnicity, n (%)**					—		.15^e^
	Chinese	45 (72.6)	48 (78.7)				
	Malay	7 (11.3)	4 (6.5)				
	Indian	4 (6.4)	0 (0)				
	Others	6 (9.7)	9 (14.8)				
**Religion, n (%)**					2.10 (3)		.55^d^
	Buddhism	21 (33.9)	27 (44.3)				
	Christianity	24 (38.7)	23 (37.7)				
	Islam	7 (11.3)	5 (8.2)				
	Others	10 (16.1)	6 (9.8)				
**Education, n (%)**					0.95 (3)		.81^d^
	Primary school or lower	7 (11.3)	10 (16.4)				
	Secondary school	24 (38.7)	20 (32.8)				
	Polytechnic or college	14 (22.6)	13 (21.3)				
	University	17 (27.4)	18 (29.5)				
**Employment, n (%)**					—		≥.99^e^
	Employs	38 (61.3)	37 (60.7)				
	Unemployed	10 (16.1)	11 (18)				
	Retired	10 (16.1)	9 (14.8)				
	Others	4 (6.5)	4 (6.5)				
**Occupation, n (%)**					—		.88^e^
	Professional or management	11 (17.7)	11 (18)				
	Sales or executive	8 (12.9)	8 (13.1)				
	Clerical or technical	10 (16.1)	10 (16.4)				
	Self-employed	5 (8.1)	2 (3.3)				
	Others	28 (45.2)	30 (49.2)				
**Monthly income (in SGD^f^), n (%)**					0.20 (2)		.91^d^
	<$3000	27 (43.5)	29 (47.5)				
	$3000-$5000	14 (22.6)	13 (21.3)				
	>$5000	21 (33.9)	19 (31.2)				
**Living with grandchildren, n (%)**					—		≥.99^e^
	Yes	5 (8.1)	4 (6.6)				
	No	57 (91.9)	57 (93.4)				
**Living with spouse, n (%)**					0.75 (1)		.38^d^
	Yes	37 (59.7)	41 (67.2)				
	No	25 (40.3)	20 (32.8)				
**Living with children, n (%)**					0.39 (1)		.53^d^
	Yes	40 (64.5)	36 (59)				
	No	22 (35.5)	25 (41)				
**Living with siblings, n (%)**					0.22 (1)		.64^d^
	Yes	10 (16.7)	8 (13.1)				
	No	52 (83.3)	53 (86.9)				
**Living with maid, n (%)**					0.00 (1)		.98^d^
	Yes	6 (9.7)	6 (9.8)				
	No	56 (90.3)	55 (90.2)				
**Type of surgery, n (%)**					—		.96^e^
	Mastectomy	34 (54.8)	35 (57.4)				
	Lumpectomy	24 (38.7)	22 (36.1)				
	Others	4 (6.4)	4 (6.5)				
**Breast cancer stage, n (%)**					—		.22^e^
	Stage 0	15 (24.2)	9 (14.8)				
	Stage 1	18 (29)	14 (22.9)				
	Stage 2	24 (38.7)	27 (44.3)				
	Stage 3	5 (8.1)	11 (18)				
**With cardiovascular disease, n (%)**					0.10 (1)		.32^d^
	Yes	21 (33.9)	26 (42.6)				
	No	41 (66.1)	35 (57.4)				
**With lung disease, n (%)**				—		.62^e^	
	Yes	1 (1.6)	2 (3.3)				
	No	61 (98.4)	59 (96.7)				
**With diabetes disease, n (%)**					—		≥.99^e^
	Yes	4 (6.4)	3 (4.9)				
	No	58 (93.6)	58 (95.1)				
**Chemotherapy, n (%)**					0.98 (1)		.32^d^
	Yes	27 (43.5)	32 (52.4)				
	No	35 (56.5)	29 (47.6)				
**Radiotherapy, n (%)**					0.41 (1)		.52^d^
	Yes	19 (30.6)	22 (36.1)				
	No	43 (69.4)	39 (63.9)				

^a^Independent sample *t* test.

^b^Not applicable.

^c^Mann-Whitney *U* test.

^d^Chi-square test.

^e^Fisher exact test.

^f^SGD $3000=~US $2276.28 and SGD $5000=~US $3793.80.

**Table 2:** Comparison of the participants’ primary and secondary outcomes between groups at baseline (n=123).

Baseline outcomes		iCareBreast group (n=62), mean (SD)	Control group (n=61), mean (SD)	*t* test (*df*)	*P* value
Perioperative self-efficacy (GSES)^a,b^		30.4 (5.5)	30.2 (4.6)	0.25 (120)	.80
Anxiety (HADS-A)^a,c^		7.6 (3.5)	8.1 (3.5)	–0.73 (120)	.47
Depression (HADS-D)^a^		4.4 (3.4)	4.5 (3.5)	–0.29 (120)	.74
Fatigue (MFSI)^a,d^		11.3 (18.7)	11.4 (20.7)	–0.05 (121)	.96
**Quality of life (QLQ-BR23)^e,f^**					
	BRBI^g^	86.4 (18.5)	80.5 (22.4)	—	.09
	BRSEF^h^	15.9 (27.4)	14.2 (17.7)	—	.39
	BRSEE^i^	42.1 (34.9)	31.9 (29.3)	—	.33
	BRFU^j^	45.2 (30.8)	42.6 (30.5)	—	.69
	BRST^k^	18.0 (18.2)	18.8 (17.8)	—	.81
	BRBS^l^	12.8 (16.4)	12.3 (14.1)	—	.07
	BRAS^m^	10.2 (13.2)	12.2 (19.9)	—	.99
	BRHL^n^	22.2 (27.6)	39.5 (35.8)	—	.05

^a^Independent samples *t* test.

^b^GSES: General Self-Efficacy Scale.

^c^HADS: Hospital Anxiety and Depression Scale.

^d^MFSI: Multidimensional Fatigue Symptom Inventory.

^e^Mann-Whitney *U* test.

^f^QLQ-BR23: Quality of Life Breast Cancer.

^g^BRBI: body imagers.

^h^BRSEF: sexual functioning.

^i^BRSEE: sexual enjoyment.

^j^BRFU: future perspective.

^k^BRST: systemic therapy side effects.

^l^BRBS: breast symptoms.

^m^BRAS: arm symptoms.

^n^BRHL: upset by hair loss.

#### Comparisons of Primary and Secondary Outcomes Between Groups at Each Posttest Timepoint

Table 3 shows no significant differences between the groups for the primary outcome at posttest 1 (immediately after the intervention or 2 weeks after surgery; T1), and posttest 2 (2.5 months after the intervention or 3 months after surgery; T2). Similarly, no significant differences were observed for any secondary outcomes at both follow-ups. The detailed analysis results are presented in Table S1 in Multimedia Appendix 3. In the intervention group, perioperative self-efficacy scores declined at posttest 1 but increased at posttest 2. In contrast, self-efficacy scores in the control group showed a continuously increasing trend from baseline to posttest 2. A linear mixed model, including a time×engagement level interaction, was conducted to compare participants with higher engagement (completion rate ≥60% of the intended use days) and lower engagement (completion rate <60% of the intended use days), following the classification method used in previous research [49]. The results indicated that engagement level did not significantly influence the perioperative self-efficacy trajectory over time (*F*_2,51.53_=1.38; *P*=.26; Table S2 in Multimedia Appendix 3).

**Table 3:** Comparisons of the primary outcome between groups at follow-up time points T1 and T2 (n=123).

Perioperative self-efficacy (GSES)^a,b^	iCareBreast group (n=62), mean (SD)	Control group (n=61), mean (SD)	Mean difference (95% CI)	*P* value
T1^c^	28.73 (5.33)	30.36 (4.54)	–1.63 (–3.43 to 0.18)	.08
T2^d^	29.42 (5.55)	31.32 (5.22)	–1.90 (–4.06 to 0.26)	.09

^a^GSES: General Self-Efficacy Scale.

^b^Independent samples *t* test.

^c^T1: immediately after the intervention (2 weeks after surgery).

^d^T2: 2.5 months after the intervention (3 months after surgery).

#### Comparison of Outcomes Within the Two Groups Over Time

A repeated measures analysis was conducted to assess the intervention’s effect on all outcomes. The overall test results are presented in Table S3 in Multimedia Appendix 3. Statistically significant time effects were observed for changes in anxiety scores (*F*_2,214.21_=24.54; *P*<.001) and fatigue scores (*F*_2,215.69_=4.21; *P*=.02), as well as across all quality-of-life subscales except for sexual enjoyment. Significant changes were noted in body image (*F*_2,220.87_=4.54; *P*=.01), sexual function (*F*_2,218.86_=4.01; *P*=.02), future perspective (*F*_2,217.11_=12.24; *P*<.001), systemic therapy side effects (*F*_2,221.70_=6.72; *P*=.001), breast symptoms (*F*_2,221.00_=38.40; *P*<.001), arm symptoms (*F*_2,218.33_=57.04; *P*<.001), and upset by hair loss (*F*_2,112.01_=3.60; *P*=.03). Additionally, a significant group×time interaction effect was observed for changes in perioperative self-efficacy scores (*F*_2,214.70_=3.93; *P*=.02).

The overall trends of primary and secondary outcomes from baseline (T0) to immediately after the intervention (T1) and 2.5 months after the intervention (T2) for participants in both groups are shown in Figures S1-S13 in Multimedia Appendix 4. Participants in the intervention group experienced a decrease in self-efficacy scores from T0 to T1 and an increase from T1 to T2. In contrast, the control group showed a continuous increase in self-efficacy from T0 through T2, with intervention group scores lower than the control group at both T1 and T2 (Figure S1 in Multimedia Appendix 4). Anxiety scores for both groups showed similar downward trends from T0 to T2 (Figure S3 in Multimedia Appendix 4). Depression scores in the intervention group continued to decrease from T0 to T2, while the control group saw an increase from T0 to T1 and a decrease at T2, with lower depression scores for the intervention group at both T1 and T2 (Figure S4 in Multimedia Appendix 4). Fatigue scores showed a downward trend for both groups at T1; however, the intervention group experienced an increase at T2, while the control group continued to show a decrease (Figure S3 in Multimedia Appendix 4). The QLQ-BR23 subscale for future perspective showed a consistent increase across the study period (Figure S9 in Multimedia Appendix 4). For trends in the remaining quality of life subscales, please refer to Multimedia Appendix 4. Perioperative satisfaction scores were higher in the intervention group than in the control group at T1 and increased further from T1 to T2, whereas the control group showed a decrease in satisfaction from T1 to T2 (Figure S13 in Multimedia Appendix 4).

#### Engagement: iCareBreast App Use

**Figure 4:**
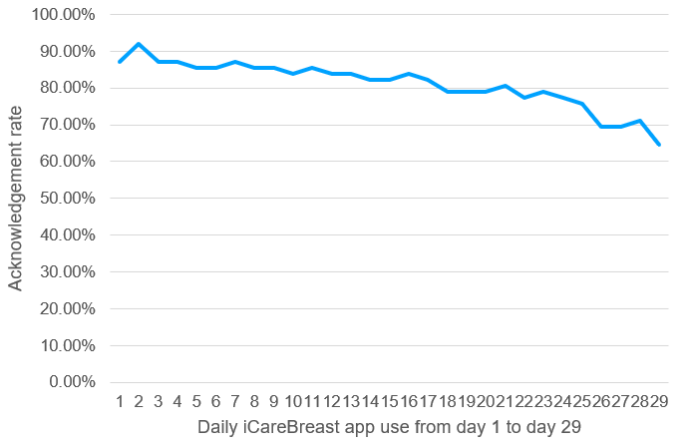
iCareBreast app acknowledgment rate (%) tracking.

Participants’ daily use of the iCareBreast app was systematically tracked, and app engagement was measured using acknowledgment rates, calculated as the proportion of users who completed their assigned daily tasks out of the 62 intended users. A progressive decline in engagement was observed, with active user participation decreasing from 99% on day 1 to below 80% by day 19 and further declining to approximately 64% by the final day of app use (day 29). The daily acknowledgment rates are presented in Figure 4.

A subgroup analysis based on app engagement levels was conducted, classifying users into high engagement (completion rate ≥60% of the intended use days) and low engagement (completion rate <60% of the intended use days). No significant differences were found between the two groups in the measured outcomes, except for depression scores at posttest 2. The mean depression score was significantly lower in the high engagement group (mean 3.61, SD 3.06) compared to the low engagement group (mean 6.29, SD 5.77; *P*=.002; 95% CI –2.66 to 8.02). Additional subgroup analysis results are presented in Table S4 in Multimedia Appendix 3.

## Discussion

### Principal Findings

To our knowledge, this is the first study to deliver timeline-based, psychosocial care to patients with early-stage breast cancer during the perioperative period. The study aimed to assess the impact of a breast cancer digital tool—the iCareBreast app—on perioperative self-efficacy, anxiety, depression, fatigue, HR-QoL, and perioperative satisfaction over time.

Baseline characteristics between participants in the intervention and control groups showed no statistically significant differences, indicating successful randomization. Our postintervention assessments revealed no significant differences between the two groups across all measured outcomes immediately after the intervention and at the 2.5-month follow-up. This contrasts with findings from a recent Cochrane review, which reported that psychological interventions for women with early-stage breast cancer reduced depression, anxiety, and mood disturbances, while also improving HR-QoL [18]. Notably, most RCTs in the review delivered interventions through face-to-face sessions, typically led by health care or research professionals. Guided digital interventions that incorporate additional contact with professionals, either in person or digitally, have been shown to be more effective in reducing distress, anxiety, and fatigue compared to nonguided (self-directed) digital interventions, which are often associated with lower patient engagement [49]. Guided psychosocial interventions generally yield greater benefits than self-guided formats, likely due to increased professional interactions that enhance perceived support, motivation, and accountability for adherence throughout the study period [50,51]. In contrast, our study relied solely on a mobile app for intervention delivery, requiring participants to engage with the content independently. This difference in delivery format may explain the variation in findings between this study and those of Jassim et al [18]. Future psychosocial and educational interventions delivered via mobile health (mHealth) platforms could benefit from incorporating live support features, such as real-time oncology nurse chat functions or direct psycho-oncologist feedback, to improve engagement and intervention effectiveness.

Our finding of no significant difference in anxiety and depression aligns with a recent meta-analysis evaluating mHealth interventions for psychological issues in women with breast cancer undergoing chemotherapy, which also showed no significant changes in these outcomes [52]. Conversely, a pilot RCT by Foley et al [53] reported that patients using an mHealth app that provided information on basic breast cancer biology and common surgery types experienced increased anxiety and depression 1 week after surgery. The authors attributed this to possible confounding factors such as fatalistic attitudes and difficulty using a tablet device. This suggests that while some patients may find mHealth tools helpful to their care, others may experience increased anxiety due to constant reminders of their condition [54].

Evidence in the literature also indicates that anxiety and depression may be more pronounced in patients with higher baseline levels of these symptoms throughout follow-up [18]. For example, a recent large-scale RCT of an app-based support tool, PINK!Coach, introduced to patients with breast cancer during treatment and used over a 3-month period, showed significant reductions in psychological distress within the intervention group. Notably, 36% (n=61) of app users during treatment reported moderate to severe depression at baseline [55]. In contrast, our study participants reported low baseline levels of anxiety and depression, which may have reduced their potential to benefit from the intervention. For patients at higher risk of anxiety—such as those who are younger, have lower education levels, or are undergoing particularly daunting procedures—additional support from a psycho-oncologist and clear communication about the treatment may be particularly beneficial [10].

While participants generally reported decreases in fatigue over time, these differences were not statistically significant. This finding contrasts with a recent review, which reported that physical exercise and psychosocial interventions reduced fatigue over a 2- to 6-month follow-up period [56]. Despite the app’s inclusion of daily physiotherapy exercises, mindfulness practices, and positive psychology techniques intended to alleviate physical and emotional fatigue, we did not observe an immediate or later reduction in fatigue. The different findings may be attributable to our intervention being app-based, requiring patients to self-practicing at home. As a result, adherence to those practices may have been lower due to the lack of close monitoring by health care professionals. Another potential reason for the lack of significant reduction in fatigue could be the relatively short duration of our intervention. Studies have shown that exercise interventions with low to moderate intensity—20 minutes per day, three times per week, over 12 weeks—can significantly reduce fatigue in patients with breast cancer [57]. Since cancer-related fatigue can persist during and beyond active treatment, ongoing monitoring and longer physiotherapy interventions could be more effective. Future tailored interventions, such as oncological physiotherapy, mindfulness, or yoga, should be customized to meet patients’ characteristics, needs, and preferences, ideally facilitated by a nurse or relevant specialist, to reduce the impact of fatigue on QoL [58].

This study found no differences in HR-QoL across all subscales, aligning with findings from a recent RCT that also reported no significant differences in quality of life between intervention and control groups [54]. In this study, most HR-QoL subscales fluctuated over time. Notably, both the intervention and control groups reported an increase in the “future perspective” subscale, which likely reflects a shared uncertainty about the future. Previous studies suggest that after surgery, patients often experience uncertainty, anxiety, and distress related to surgery outcomes, the need for additional therapies, and the possibility of recurrence. Many patients endure prolonged distress as they wait several weeks for postoperative appointments to discuss these concerns [12]. Additionally, half of our participants received chemotherapy, and one-third underwent radiation therapy during the study period. Since the iCareBreast app primarily focused on perioperative care, it may not have fully addressed participants’ needs or provided sufficient information on chemotherapy and radiotherapy. This support gap may have further heightened their concerns about the future.

Finally, participants in the intervention group reported an increasing trend in perioperative care satisfaction compared to the control group. This aligns with findings from a recent systematic review, which reported high satisfaction levels among participants using eHealth platforms for breast cancer supportive care interventions, suggesting that mHealth may be an acceptable approach for patients due to accessibility and convenience [19].

Patient engagement with the mobile app is a critical factor influencing its effectiveness. Although the app was designed to provide comprehensive resources—including education on breast cancer diagnosis and treatment, psychosocial support, a digital interface for questions, daily app push notifications, and web-based dashboard reminders—active use and engagement were not guaranteed. Despite incorporating engagement measures such as daily reminders from the app, additional prompts from researchers via the web-based dashboard, and a chat feature for posting concerns or queries for health care providers to address, these methods were insufficient to sustain user engagement. A progressive decline in app use was observed, which may have limited its impact on patient-reported outcomes. This trend aligns with prior studies suggesting that declining engagement in digital health interventions is often linked to reduced motivation over time [54]. Although subgroup analysis showed no significant differences in most outcomes between high and low-engagement groups (using a 60% adherence rate as the cutoff), a lack of sustained engagement remains a key challenge in digital health interventions. Evidence suggests that patients who engage more actively with digital health tools derive greater benefits in managing their health [59]. Future mHealth interventions may benefit from enhanced engagement strategies, such as incorporating reward systems, involving facilitators, integrating patient-preferred features, and using a participatory design approach. Allowing patients to contribute to content development tailored to their treatment stage and psychological needs may further promote sustained engagement and improve intervention effectiveness [49,59-61].

### Lessons Learned

This study underscores the importance of guidance, adherence, and engagement in mHealth interventions for patients with breast cancer. Guided digital interventions with direct health care or research professional interactions are more effective than self-guided formats, suggesting that features like live nurse chat or psycho-oncologist feedback could enhance efficacy. Additionally, adherence to self-guided exercise content was limited without supervision, emphasizing the need for behavioral reinforcement and provider involvement. Finally, declining app engagement suggests that passive reminders alone are insufficient—future interventions should incorporate user-responsive features, gamification, and participatory content design to sustain long-term use.

### Strengths and Limitations

Several limitations in this psychosocial intervention RCT for patients with breast cancer warrant consideration. First, due to the intervention’s nature, blinding participants was not feasible, which may have introduced performance bias; however, the extent of its impact on treatment effects is uncertain. Second, the study’s data were collected from a single public hospital, and participants needed to know how to use a mobile app. This may limit the generalizability of the results to a broader patient population. Furthermore, individuals with pre-existing anxiety, stress, or depressive disorders who were undergoing active treatment were excluded, despite being more likely to benefit from the intervention. Finally, the follow-up period was limited to 2.5 months after surgery, which may not have been sufficient to capture the long-term effects of the intervention. Given that mHealth interventions often require sustained engagement and behavioral adaptation, a longer follow-up period (eg, 6-12 months) may be necessary to fully evaluate the intervention’s long-term efficacy.

### Implications for Practice and Recommendations for Future Research

The normalization of technology use, particularly accelerated by the COVID-19 pandemic, has become a routine part of daily life. Digital tools for delivering psychological interventions hold promise for improving psychological well-being and HR-QoL in women with early-stage breast cancer. Health care professionals should consider addressing the individual care needs of patients with breast cancer during active treatment periods, such as the perioperative phase. Future research should focus on enhancing mHealth engagement through user-responsive strategies such as real-time professional support, peer communities, and gamification. Guided interventions, including live nurse chats or psycho-oncologist consultations, may improve outcomes over self-guided formats. Studies should also explore personalized content tailored to patient characteristics and consider targeting women with clinically significant levels of anxiety and depression to evaluate the potential clinical benefits of psychosocial interventions in this subgroup. Additionally, research should evaluate the long-term benefits beyond the perioperative period to assess sustained effects.

### Conclusions

This study evaluated the mobile-based perioperative care program, iCareBreast, for women undergoing breast cancer surgery. Compared to usual care alone, the intervention did not show significant effects on health outcomes or HR-QoL. However, participants using the iCareBreast reported higher perioperative care satisfaction scores than the control group, which underscores the acceptability of mHealth tools in oncological settings. These findings highlight the need for further refinement of the program, offering valuable insights for optimizing iCareBreast’s effectiveness in improving perceived health outcomes. Future research should focus on enhancing patient engagement strategies and exploring personalized approaches to better meet the perioperative care needs of patients with breast cancer.

## Appendix

Multimedia Appendix 1 CONSORT eHEALTH checklist (V 1.6.1).

Multimedia Appendix 2 Summary of the day to day content of iCareBreast.

Multimedia Appendix 3 Comparisons of outcomes between groups.

Multimedia Appendix 4 Comparison of outcomes variables changes over time between groups.

## Abbreviations

CONSORT: Consolidated Standards of Reporting Trials
HR-QoL: health-related quality of life
MD: mean difference
mHealth: mobile health
RCT: randomized controlled trial

## Data Availability

The datasets generated or analyzed during this study are available from the corresponding author upon reasonable request.
