# Supplementary material for: Effectiveness of an Innovative Mobile-Based Perioperative Care Program for Women Undergoing Breast Cancer Surgery (iCareBreast): Randomized Controlled Trial
Source: J Med Internet Res. 2025 Apr 21;27:e71684. doi: 10.2196/71684 (PMC12053255; doi:10.2196/71684)
Supplement: Multimedia Appendix 2 [file jmir_v27i1e71684_app2.docx]

| iCareBreast | | |
| --- | --- | --- |
|  | Time-point | Information |
| Pre-operation | Pre-operative day 14 | 1. Welcome message 2. Pre-operative checklist 3. Pre-operative preparation 4. Financial Counselling 5. Motivational quote and mindfulness-based practice |
|  | Pre-operative day 13 | 1. After your diagnosis 2. Motivational quote |
|  | Pre-operative day 12 | 1. Anatomy of the breasts and surrounding organs 2. Overview of breast cancer surgery 3. Emotions 4. Motivational quote and mindfulness-based practice |
|  | Pre-operative day 11 | 1. Pre-admission/ Anaesthesia assessment 2. Social support 3. Motivational quote and mindfulness-based practice |
|  | Pre-operative day 10 | 1. Deep breathing techniques 2. Motivational quote and mindfulness-based practice |
|  | Pre-operative day 9 | 1. Relaxation 2. Motivational quote and mindfulness-based practice |
|  | Pre-operative day 8 | 1. Positive reinforcement 2. Motivational quote and mindfulness-based practice |
|  | Pre-operative day 7 | 1. Surgery preparation 2. Distraction 3. Motivational quote and mindfulness-based practice |
|  | Pre-operative day 6 | 1. Physiotherapy: Raised-arm exercise 2. Motivational quote and mindfulness-based practice |
|  | Pre-operative day 5 | 1. Physiotherapy: Wand exercise 2. Motivational quote and mindfulness-based practice |
|  | Pre-operative day 4 | 1. Physiotherapy: Shoulder mobility exercise 2. Motivational quote and mindfulness-based practice |
|  | Pre-operative day 3 | 1. Physiotherapy: Chest wall stretch 2. Motivational quote and mindfulness-based practice |
|  | Pre-operative day 2 | 1. Preparing for the surgery 2. Motivational quote and mindfulness-based practice |
|  | Pre-operative day 1 | 1. Pre-operative instructions 2. Anaesthesia 3. Lymphoscintigraphy 4. Intra-operative information 5. Motivational quote and mindfulness-based practice |
| Operation day | Operation day | 1. Recovery after surgery 2. Motivational quote |
|  | Post-operative day 1 | 1. Wound management 2. Cannula and drain care 3. Pain control 4. Pop-up reminder  - Physiotherapy - Non-pharmacological methods for pain relief, such as deep breathing, relaxation, positive reinforcement, and distraction - Motivational quote and mindfulness-based practice |
|  | Post-operative day 2 | 1. Breast prosthesis 2. Social support 3. Discharge information and follow up 4. Pop-up reminder  - Physiotherapy - Non-pharmacological methods for pain relief, such as deep breathing, relaxation, positive reinforcement, and distraction - Motivational quote and mindfulness-based practice |
|  | Post-operative day 3 | 1. Signs of wound infection 2. Drain care 3. Pop-up reminder  - Physiotherapy - Non-pharmacological methods for pain relief, such as deep breathing, relaxation, positive reinforcement, and distraction - Motivational quote and mindfulness-based practice |
|  | Post-operative day 4 | 1. Other social support group 2. Pop-up reminder  - Physiotherapy - Non-pharmacological methods for pain relief, such as deep breathing, relaxation, positive reinforcement, and distraction - Motivational quote and mindfulness-based practice |
|  | Post-operative day 5 | 1. Pop-up reminder  - Physiotherapy - Non-pharmacological methods for pain relief, such as deep breathing, relaxation, positive reinforcement, and distraction - Motivational quote and mindfulness-based practice |
|  | Post-operative day 6 | 1. Emotional support 2. Pop-up reminder  - Physiotherapy - Non-pharmacological methods for pain relief, such as deep breathing, relaxation, positive reinforcement, and distraction - Motivational quote and mindfulness-based practice |
|  | Post-operative day 7 | 1. Pop-up reminder  - Physiotherapy - Non-pharmacological methods for pain relief, such as deep breathing, relaxation, positive reinforcement, and distraction - Motivational quote and mindfulness-based practice |
|  | Post-operative day 8 | 1. Pop-up reminder  - Physiotherapy - Non-pharmacological methods for pain relief, such as deep breathing, relaxation, positive reinforcement, and distraction - Motivational quote and mindfulness-based practice |
|  | Post-operative day 9 | 1. Pop-up reminder  - Physiotherapy - Non-pharmacological methods for pain relief, such as deep breathing, relaxation, positive reinforcement, and distraction - Motivational quote and mindfulness-based practice |
|  | Post-operative day 10 | 1. Seroma aspiration 2. Pop-up reminder  - Physiotherapy - Non-pharmacological methods for pain relief, such as deep breathing, relaxation, positive reinforcement, and distraction - Motivational quote and mindfulness-based practice |
|  | Post-operative day 11 | 1. Pop-up reminder  - Physiotherapy - Non-pharmacological methods for pain relief, such as deep breathing, relaxation, positive reinforcement, and distraction - Motivational quote and mindfulness-based practice |
|  | Post-operative day 12 | 1. Pop-up reminder  - Physiotherapy - Non-pharmacological methods for pain relief, such as deep breathing, relaxation, positive reinforcement, and distraction - Motivational quote and mindfulness-based practice |
|  | Post-operative day 13 | 1. Pop-up reminder  - Physiotherapy - Non-pharmacological methods for pain relief, such as deep breathing, relaxation, positive reinforcement, and distraction - Motivational quote and mindfulness-based practice |
|  | Post-operative day 14 | 1. Arm sensation and movement 2. Permanent breast prosthesis 3. Pop-up reminder  - Physiotherapy - Non-pharmacological methods for pain relief, such as deep breathing, relaxation, positive reinforcement, and distraction - Motivational quote and mindfulness-based practice  1. Thank you message |
