# Supplementary material for: Effectiveness of an Innovative Mobile-Based Perioperative Care Program for Women Undergoing Breast Cancer Surgery (iCareBreast): Randomized Controlled Trial
Source: J Med Internet Res. 2025 Apr 21;27:e71684. doi: 10.2196/71684 (PMC12053255; doi:10.2196/71684)
Supplement: Multimedia Appendix 3 [file jmir_v27i1e71684_app3.docx]

Table S1: Comparisons of secondary outcomes between groups at follow-up time points T1 & T2 (n=123)

| Outcomes | Intervention group (n=62) | Control group (n=61) | Mean Difference (95%CI) | *P*-value |
| --- | --- | --- | --- | --- |
| **Secondary outcomes (mean, SD)** |  |  |  |  |
| Anxiety (HADS-A)^a^ |  |  |  |  |
| T1 | 6.73(3.12) | 6.39(3.65) | 0.34(-0.90 to 1.58) | .59 |
| T2 | 5.56(3.51) | 5.83(3.41) | -0.27(-1.67 to 1.13) | .70 |
| Depression (HADS-D)^a^ |  |  |  |  |
| T1 | 4.25(3.36) | 5.25(3.23) | -1.00(-2.20 to 0.20) | .10 |
| T2 | 4.02(3.66) | 4.11(3.04) | -0.09(-1.44 to 1.26) | .89 |
| Fatigue (MFSI)^a^ |  |  |  |  |
| T1 | 6.29(16.48) | 8.75(17.58) | -2.46(-8.67 to 3.76) | .44 |
| T2 | 6.84(16.94) | 7.17(19.28) | -0.33(-7.70 to 7.04) | .93 |
| **Quality of life (QLQ-BR23)^b^** |  |  |  |  |
| Functional scales |  |  |  |  |
| Body Image (BRBI) |  |  |  |  |
| T1 | 78.67(23.02) | 79.10(21.63) | -0.42(-8.57 to 7.72) | .96 |
| T2 | 75.00(24.81) | 79.97(19.82) | -4.97(-13.97 to 4.03) | .34 |
| Sexual function (BRSEF) |  |  |  |  |
| T1 | 9.04(19.65) | 11.30(18.16) | -2.26(-9.16 to 4.64) | .26 |
| T2 | 13.70(23.11) | 15.03(20.88) | -1.33(-10.24 to 7.59) | .50 |
| Sexual enjoyment (BRSEE) |  |  |  |  |
| T1 | 19.30(32.04) | 17.65(26.66) | 1.65(-18.45 to 21.75) | .98 |
| T2 | 33.33(30.86) | 35.29(27.56) | -1.96(-23,05 to 19.13) | .77 |
| Future perspective (BRFU) |  |  |  |  |
| T1 | 50.28(28.62) | 43.50(31.11) | 6.78(-4.12 to 17.68) | .19 |
| T2 | 57.04(26.23) | 58.33(28.68) | -1.30(-12.44 to 9.85) | .80 |
| Symptom scales |  |  |  |  |
| Systemic therapy side effects (BRST) |  |  |  |  |
| T1 | 14.69(11.02) | 16.38(15.97) | -1.69(-6.70 to 3.31) | .86 |
| T2 | 22.96(17.12) | 20.88(18.58) | 2.08(-5.16 to 9.33) | .43 |
| Breast symptoms (BRBS) |  |  |  |  |
| T1 | 28.67(19.22) | 25.42(18.47) | 3.25(-3.62 to 10.12) | .37 |
| T2 | 18.70(20.65) | 17.65(14.59) | 1.06(-6.13 to 8.24) | .63 |
| Arm symptoms (BRAS) |  |  |  |  |
| T1 | 28.62(23.27) | 34.28(25.47) | -5.65(-14.54 to 3.24) | .16 |
| T2 | 18.02(18.99) | 21.13(22.47) | -3.11(-11.60 to 5.39) | .55 |
| Upset by hair loss (BRHL) **^c^** |  |  |  |  |
| T1 | 13.33(24.51) | 10.93(18.97) | -0.05(-21.52 to 11.41) | .81 |
| T2 | 23.26(32.15) | 20.24(28.20) | 3.02(-9.04 to 15.08) | .65 |
| Perioperative care satisfaction (ODS) |  |  |  |  |
| T1 | 4.91(0.85) | 4.83(1.29) | 0.09(-0.32 to 0.49) | .68 |
| T2 | 4.95(0.90) | 4.61(0.96) | 0.35(0.04 to 0.73) | .08 |

Note: HADS: Hospital Anxiety and Depression Scale; MFSI: [Multidimensional Fatigue Symptom Inventory](https://www.sralab.org/rehabilitation-measures/multidimensional-fatigue-symptom-inventory-short-form#:~:text=The%20MFSI%2DSF%20consists%20of,)%20to%204%20(extremely).); QLQ-BR23: Quality of Life Breast Cancer; BRBI: Body Image; BRSEE: Sextual functioning; BRSEE: Sextual Enjoyment; BRFU: Future Perspective; BRST: Systemic therapy side effects; BRBS: Breast symptoms; BRAS: Arm symptoms; BRHL: Upset by hair loss; ODS: Ordinal Descriptive Scale; ^ₐ^: Independent samples test; ^b^: Mann-Whitney U Test; ^c^: Percent changes was used; T1: immediately post-intervention(2 weeks post-surgery); T2: 2.5 months post-intervention (3 months post-surgery).

Table S2: Subgroup analysis comparing primary outcome (perioperative self-efficacy score) changes over time between the high and low app engagement groups (n = 62)

| Outcomes | Effects | Denominator df | *F*-value | *P*-value |
| --- | --- | --- | --- | --- |
|  |  |  |  |  |
|  | Group | 60.98 | 0.61 | .44 |
|  | Time | 51.53 | 3.07 | .06 |
| Self-efficacy (GSES)^a^ | Interaction effect (group*time) | 51.53 | 1.38 | .26 |

Note: ^ₐ^: Linear mixed model

Table S3: Comparisons of outcome variables change over time between the two groups (n=123)

| Outcomes | Effects | Denominator df | *F*-value | *P*-value |
| --- | --- | --- | --- | --- |
|  |  |  |  |  |
|  | Group | 121.16 | 1.22 | .27 |
|  | Time | 214.70 | 2.34 | .10 |
| Self-efficacy (GSES) | Interaction effect (group*time) | 214.70 | 3.93 | .02 |
|  |  |  |  |  |
|  | Group | 120.43 | 0.24 | .62 |
|  | Time | 214.21 | 24.54 | <.001 |
| Anxiety (HADS-A) | Interaction effect (group*time) | 214.21 | 1.31 | .27 |
|  |  |  |  |  |
|  | Group | 120.57 | 1.10 | .30 |
|  | Time | 213.35 | 2.09 | .13 |
| Depression (HADS-D) | Interaction effect (group*time) | 213.35 | 2.13 | .12 |
|  |  |  |  |  |
|  | Group | 121.40 | 0.22 | .64 |
|  | Time | 215.69 | 4.21 | .02 |
| Fatigue (MFSI) | Interaction effect (group*time) | 215.69 | 0.36 | .70 |
|  |  |  |  |  |
|  |  |  |  |  |
|  | Group | 124.99 | 0.07 | .79 |
|  | Time | 220.87 | 4.54 | .01 |
| Body Image (BRBI) | Interaction effect (group*time) | 220.87 | 2.60 | .08 |
|  |  |  |  |  |
|  | Group | 124.74 | 0.14 | .71 |
|  | Time | 218.86 | 4.01 | .02 |
| Sexual function (BRSEF) | Interaction effect (group*time) | 218.86 | 0.94 | .39 |
|  |  |  |  |  |
|  | Group | 65.09 | 0.17 | .68 |
|  | Time | 66.89 | 3.23 | .05 |
| Sexual enjoyment (BRSEE) | Interaction effect (group*time) | 66.89 | 0.37 | .70 |
|  |  |  |  |  |
|  | Group | 121.13 | 0.62 | .43 |
|  | Time | 217.11 | 12.24 | <.001 |
| Future perspective (BRFU) | Interaction effect (group*time) | 217.11 | 0.72 | .49 |
|  |  |  |  |  |
|  | Group | 123.59 | 0.01 | .94 |
|  | Time | 221.70 | 6.72 | .001 |
| Systemic therapy side effects (BRST) | Interaction effect (group*time) | 221.70 | 0.56 | .57 |
|  |  |  |  |  |
|  | Group | 124.51 | 0.23 | .64 |
|  | Time | 221.00 | 38.40 | <.001 |
| Breast symptoms (BRBS) | Interaction effect (group*time) | 221.00 | 0.43 | 0.65 |
|  |  |  |  |  |
|  | Group | 122.90 | 1.43 | .23 |
|  | Time | 218.33 | 57.04 | <.001 |
| Arm symptoms (BRAS) | Interaction effect (group*time) | 218.33 | 0.41 | .67 |
|  |  |  |  |  |
|  | Group | 90.24 | 0.31 | .58 |
|  | Time | 112.01 | 3.60 | .03 |
| Upset by hair loss (BRHL) | Interaction effect (group*time) | 112.01 | 2.32 | .10 |
|  | Group | 110.31 | 2.04 | .16 |
|  | Time | 107.10 | 0.49 | .49 |
| Perioperative care satisfaction (ODS)^a^ | Interaction effect (group*time) | 107.10 | 0.94 | .34 |

Note: GSES: General Self-Efficacy Scale; HADS: Hospital Anxiety and Depression Scale; MFSI: [Multidimensional Fatigue Symptom Inventory](https://www.sralab.org/rehabilitation-measures/multidimensional-fatigue-symptom-inventory-short-form#:~:text=The%20MFSI%2DSF%20consists%20of,)%20to%204%20(extremely).); QLQ-BR23: Quality of Life Breast Cancer; BRBI: Body Imagers; BRSEF: Sexual functioning; BRSEE: Sexual Enjoyment; BRFU: Future Perspective; BRST: Systemic therapy side effects; BRBS: Breast symptoms; BRAS: Arm symptoms; BRHL: Upset by hair loss; ODS: Ordinal Descriptive Scale; ^a^:Post Test 1 & 2 only.

Table S4: Subgroup analyses comparing primary and secondary outcomes between participants with high and low app engagement levels at follow-up time points T1 and T2 (n = 62)

| Outcomes | Low app engagement group (n=13) | High app engagement group (n=49) | Mean Difference (95%CI) | *P*-value |
| --- | --- | --- | --- | --- |
| **Primary outcome (mean, (SD)/ median (IQR))** |  |  |  |  |
| Perioperative Self-efficacy (GSES) |  |  |  |  |
| T1 | - | 31.00(1.75) | - | .57 **^a^** |
| T2 | - | 7.00(6.00) | - | .13 **^a^** |
| **Secondary outcomes (mean, SD)** |  |  |  |  |
| Anxiety (HADS-A) |  |  |  |  |
| T1 | 6.08(3.06) | 6.89(3.15) | 0.81(-2.84 to 1.22) | .87**^b^** |
| T2 | 5.86(4.06) | 5.50(3.45) | 0.36(-2.58 to 3.30) | .97^b^ |
| Depression (HADS-D) |  |  |  |  |
| T1 | 5.86(4.06) | 5.50(3.45) | 0.36(-2.58 to 3.30) | .97^b^ |
| T2 | 6.29(5.77) | 3.61(3.06) | 2.68(-2.66 to 8.02) | **.002^b^** |
| Fatigue (MFSI)^a^ |  |  |  |  |
| T1 | - | -13(18.50) | - | .36 **^a^** |
| T2 | 8.00(13.54) | 6.63(17.64) | 1.37(-12.84 to 15.58) | .26^b^ |
| **Quality of life (QLQ-BR23)^b^** |  |  |  |  |
| Functional scales |  |  |  |  |
| Body Image (BRBI) |  |  |  |  |
| T1 | - | 25.00(58.33) | - | .18 **^a^** |
| T2 | - | - | - | .55 **^a^** |
| Sexual function (BRSEF) |  |  |  |  |
| T1 | - | 25.00(58.33) | - | .69 **^a^** |
| T2 | - | 33.33(33.33) | - | .72 **^a^** |
| Sexual enjoyment (BRSEE) |  |  |  |  |
| T1 | - | 0.00(58.33) | - | .58 **^a^** |
| T2 | - | 16.67(58.33) | - | .22 **^a^** |
| Future perspective (BRFU) |  |  |  |  |
| T1 | - | 66.67(25.00) | - | .06 **^a^** |
| T2 | - | 16.67(58.33) | - | .43 **^a^** |
| Symptom scales |  |  |  |  |
| Systemic therapy side effects (BRST) |  |  |  |  |
| T1 | - | 11.90(13.10) | - | .11 **^a^** |
| T2 | - | 16.67(28.57) | - | .87 **^a^** |
| Breast symptoms (BRBS) |  |  |  |  |
| T1 | - | 33.33(12.50) | - | .97 **^a^** |
| T2 | - | 16.67(37.50) | - | .64 **^a^** |
| Arm symptoms (BRAS) |  |  |  |  |
| T1 | - | 16.67(11.11) | - | .43 **^a^** |
| T2 | - | 11.11(19.44) | - | .18 **^a^** |
| Upset by hair loss (BRHL)^c^ |  |  |  |  |
| T1 | - | 0.00(25.00) | - | .22 **^a^** |
| T2 | - | 33.33(33.33) | - | .90 **^a^** |
| Perioperative care satisfaction (ODS) |  |  |  |  |
| T1 | - | 5.00(1.00) | - | .48 **^a^** |
| T2 | - | 5.00(0.00) | - | .44 **^a^** |

Note: GSES: General Self-Efficacy Scale; HADS: Hospital Anxiety and Depression Scale; MFSI: [Multidimensional Fatigue Symptom Inventory](https://www.sralab.org/rehabilitation-measures/multidimensional-fatigue-symptom-inventory-short-form#:~:text=The%20MFSI%2DSF%20consists%20of,)%20to%204%20(extremely).); QLQ-BR23: Quality of Life Breast Cancer; BRBI: Body Image; BRSEE: Sextual functioning; BRSEE: Sextual Enjoyment; BRFU: Future Perspective; BRST: Systemic therapy side effects; BRBS: Breast symptoms; BRAS: Arm symptoms; BRHL: Upset by hair loss; ODS: Ordinal Descriptive Scale; ^a^: Mann-Whitney U Test ^b^: Independent samples test; ^c^: Percent changes was used; T1: immediately post-intervention(2 weeks post-surgery); T2: 2.5 months post-intervention (3 months post-surgery).
