# Supplementary material for: Effectiveness of an Innovative Mobile-Based Perioperative Care Program for Women Undergoing Breast Cancer Surgery (iCareBreast): Randomized Controlled Trial
Source: J Med Internet Res. 2025 Apr 21;27:e71684. doi: 10.2196/71684 (PMC12053255; doi:10.2196/71684)

Figure S1: Means of general self-efficacy score over time between two groups


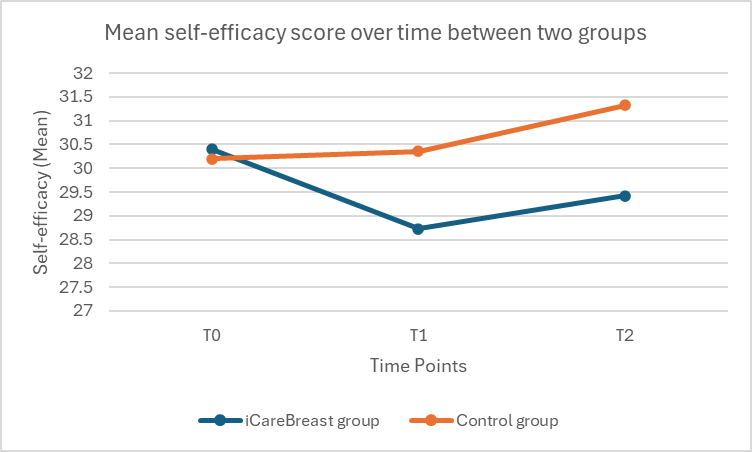


Figure S2: Means of anxiety score over time between two groups


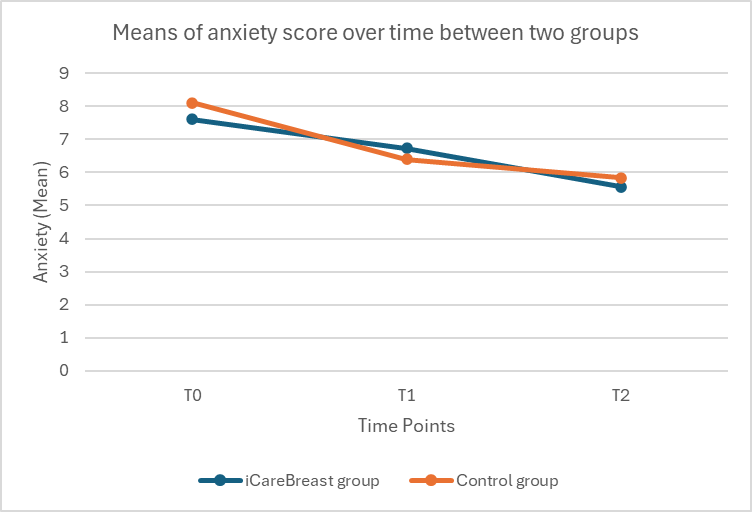


Figure S3: Means of depression score over time between two groups
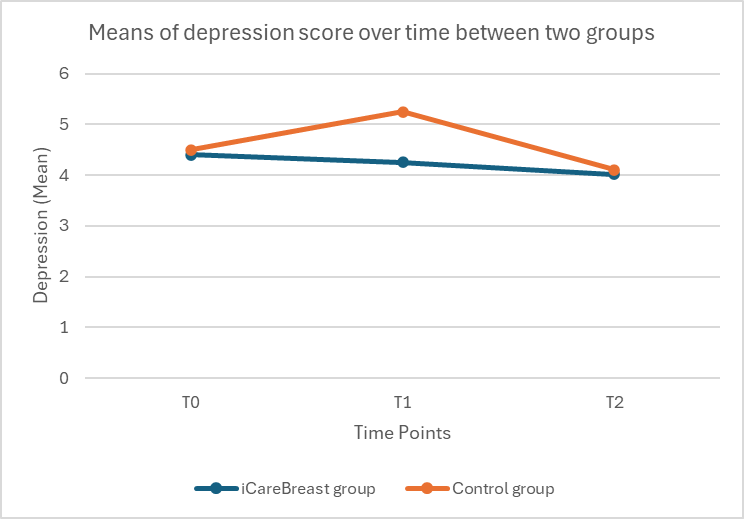


Figure S4: Means of Fatigue score over time between two groups

Figure S5: Means of body image score over time between two groups


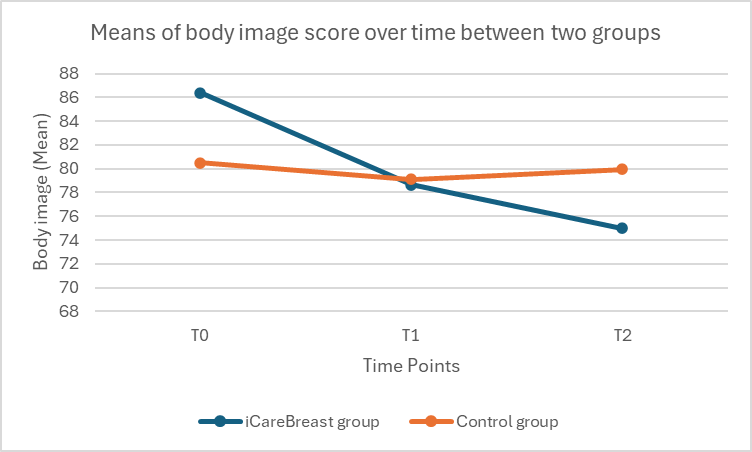


Figure S6: Means of Sexual function score over time between two groups


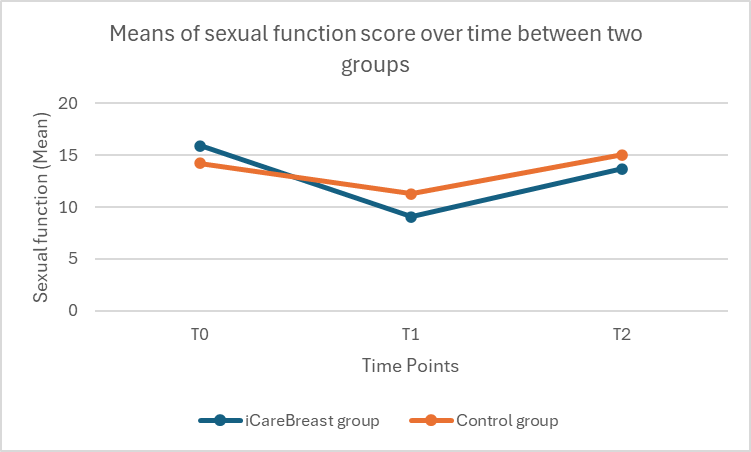


Figure S7: Means of Sexual enjoyment score over time between two groups


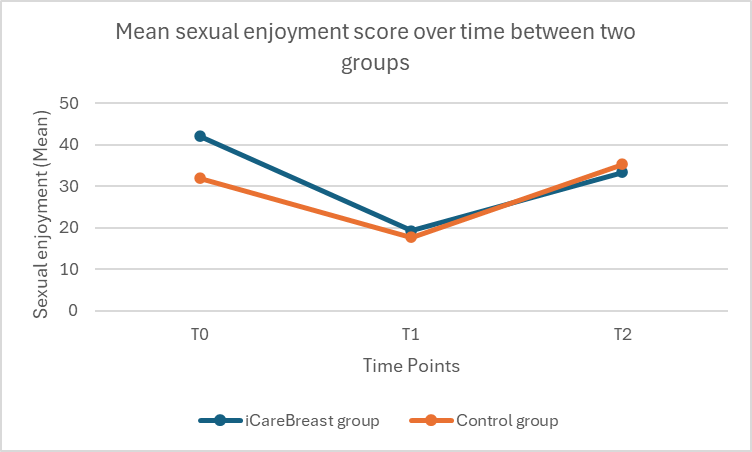


Figure S8: Means of Future perspective score over time between two groups


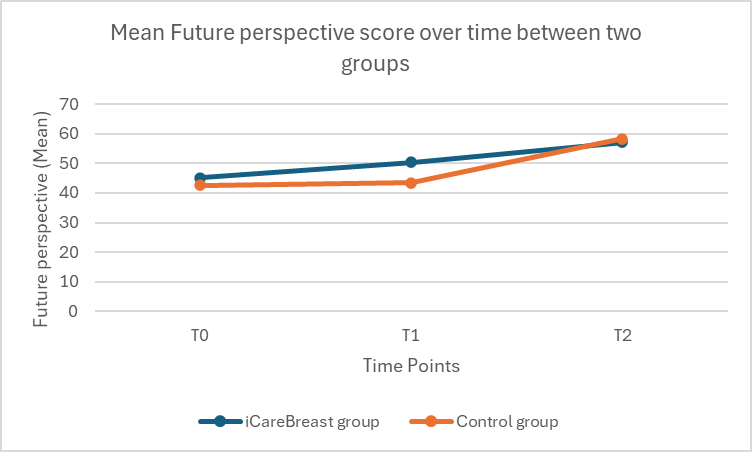


Figure S9: Means of Systemic therapy side effects score over time between two groups


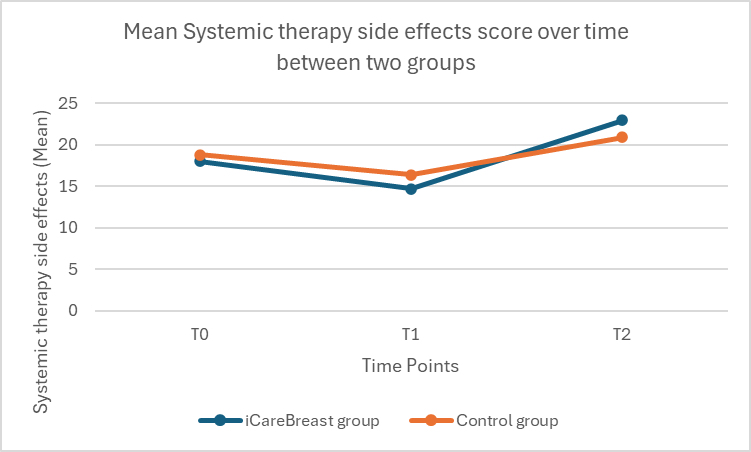


Figure S10: Means of Breast symptoms score over time between two groups


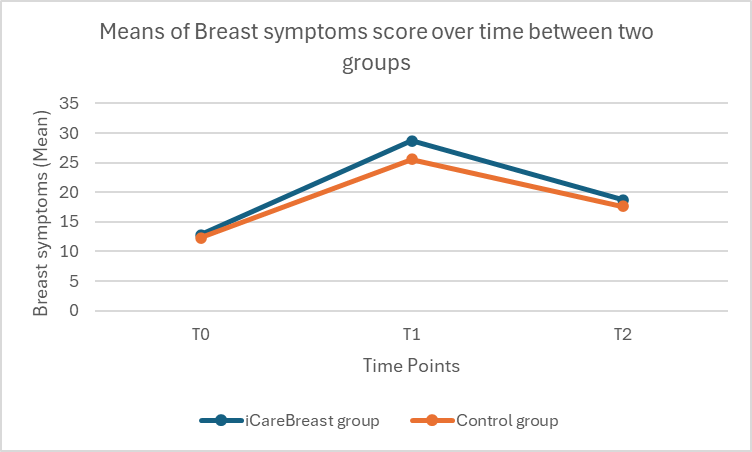


Figure S11: Means of Arm symptoms score over time between two groups


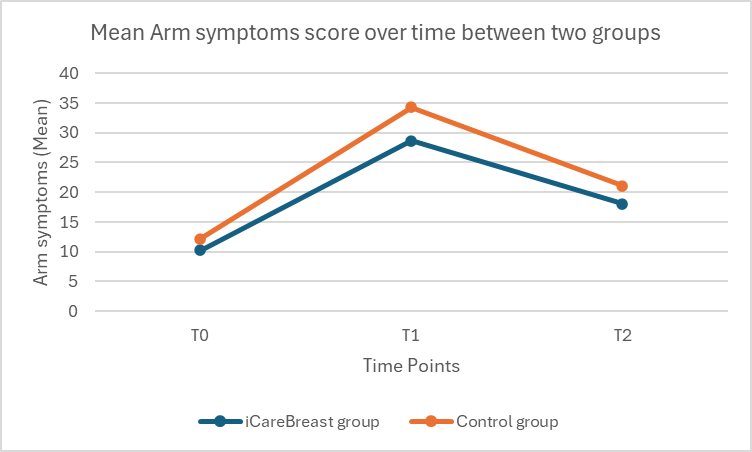


Figure S12: Means of Upset by hair loss score over time between two groups


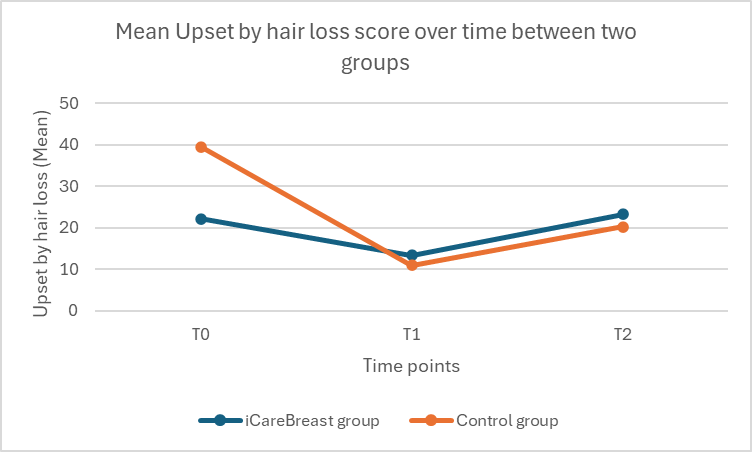


Figure S13: Means of Perioperative satisfaction score over time (Post intervention 1 & 2) between two groups


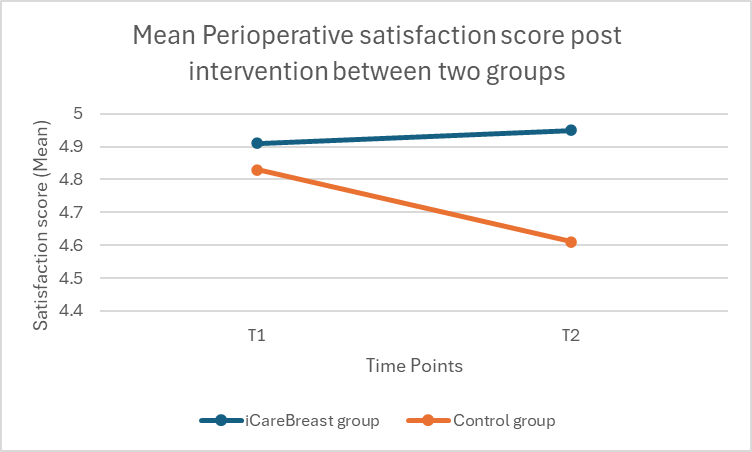

Supplement: Multimedia Appendix 4 [file jmir_v27i1e71684_app4.docx]
